# Synthetic Musk Compounds and Effects on Human Health?

**DOI:** 10.1289/ehp.113-a802b

**Published:** 2005-12

**Authors:** Daniel Salvito

**Affiliations:** Research Institute for Fragrance Materials, Inc., Woodcliff Lake, New Jersey, E-mail: dsalvito@rifm.org

A recent article by Luckenbach and Epel (2005) on *in vitro* observations of inhibitory properties exhibited by certain nitromusk and polycyclic musk fragrance ingredients on mussel cells raised some concerns regarding potential environmental risks and safety to humans that may be associated with nitromusk and polycyclic musk compounds. The Research Institute for Fragrance Materials would like to address several points that may help readers more clearly understand the meaning and context of the reported research.

The tonnages of musk compounds reported by Luckenbach and Epel (2005) in their article (7,000–8,000 tons) are higher than the industry-reported global tonnage of these materials. From 1995 to 2000, the total worldwide usage declined from 300 tons to 200 tons for musk xylene and musk ketone combined. The 2000 worldwide use of polycyclic musks is approximately 4,000 tons.

Measured concentrations of these compounds in the environment are less than the effects concentrations reported by Luckenbach and Epel (2005). In a review of measured environmental concentrations, Rimkus (1999) stated that the highest reported measurement of hexahydrohexamethyl-cyclopenta (γ)-2-benzopyran (HHCB) in surface water was 12.5 μg/L (0.048 μM). The IC_50_ (concentration that inhibits 50%) reported for polycyclic musks was 2.34 μM. Overall, measured environmental concentrations were 2–6 orders of magnitude lower than the effects concentrations reported by Luckenbach and Epel (2005).

The data reported by Luckenbach and Epel (2005) reflect a method under development. There are many steps between the observation of an *in vitro* effect and effects on whole organisms, ecosystems, and humans. *In vivo* studies in mussels and studies linking mussel gill tissue to undefined tissues in mammals and humans are some of the research necessary to conclude that these higher level effects may exist. These effects would then need to be placed into a risk-based context by comparing them to exposure concentrations.

The safety of nitromusk and polycyclic musk compounds for humans has been extensively tested and affirmed by numerous regulatory agencies and academic scientists around the world [Scientific Committee on Cosmetic Products and Non-Food Products (SCCNFP) 2002a, 2002b]. The trace environmental levels of the musks continue to be investigated, and environmental safety and monitoring studies are ongoing so that the public can be assured of their safety.

Regarding the environmental effects of synthetic musks, the IC_10_ (concentration that inhibits 10%) values should be compared to no observed effect concentrations (NOECs). The IC_10_ values of the synthetic musks are around the level of the lowest *in vivo* NOECs observed for aquatic organisms.

Table 1 shows that the *in vitro* multi-xenobiotic resistance (MXR) transporter activity in mussel gill is of the same sensitivity as the effects observed in the standard toxicity tests with aquatic organisms. Thus, at the exposure level where the protective transporter efflux is decreased, rendering the cell more accessible to other potential toxicants, the effects of the synthetic musks are also indicated in other end points, such as development and growth.

The observed effects are not limited to the nitromusk and polycyclic musk compounds. For example, the other chemicals used by Luckenbach and Epel (2005)—verapamil and quinidine—also produced the phenomenon. In the case of verapamil, the IC_10_ was reported at 1–2 orders of magnitude below the nitromusks and polycyclic musks.

I look forward to continued discussions with the Luckenbach and Epel to determine the relevance of the results of this study.

## Figures and Tables

**Table 1:** Intensity of *in vitro* MXR transporter activity in mussel gill.

	IC10) (mg/L)	Lowest NOEC (mg/L
MK	0.14 mmol = 0.041	0.063
MX	0.09 mmol = 0.027	0.056
AHTN	0.35 mmol = 0.090	0.035
HHCB	0.37 mmol = 0.095	0.068

Abbreviations: MK, musk ketone; MX, musk xylene.
